# Burnout syndrome and work engagement in nursing staff: a systematic review and meta-analysis

**DOI:** 10.3389/fmed.2023.1125133

**Published:** 2023-07-17

**Authors:** Miguel Ángel Vargas-Benítez, Francisco José Izquierdo-Espín, Nuria Castro-Martínez, José L. Gómez-Urquiza, Luis Albendín-García, Almudena Velando-Soriano, Guillermo A. Cañadas-De la Fuente

**Affiliations:** ^1^San Cecilio Clinical University Hospital, Granada, Spain; ^2^Critical Care Unit, General University Hospital of Jaén, Jaén, Spain; ^3^Internal Medicine Service, General University Hospital of Jaen, Jaén, Spain; ^4^School of Health Sciences, University of Granada, Cortadura del Valle s/n, Ceuta, Spain; ^5^Casería de Montijo Health Centre, Granada-Metropolitan Health District, Granada, Spain; ^6^Instituto de Investigación Biosanitaria (ibs.GRANADA), Granada, Spain; ^7^School of Health Sciences, University of Granada, Granada, Spain; ^8^Brain, Mind and Behaviour Research Centre (CIMCYC), University of Granada, Granada, Spain

**Keywords:** burnout, work engagement, critical care, nursing, nurses staff

## Abstract

**Background:**

A difficult and demanding work environment, such as that often experienced in healthcare, can provoke fatigue, anxiety, distress, and discomfort. This study considers factors that may influence levels of burnout and work engagement among nurses and seeks to determine the relationship between these conditions.

**Method:**

A systematic scoping review was performed, in accordance with the PRISMA Extension for Scoping Reviews, based on data obtained from a search of the PubMed/MEDLINE and Scopus databases carried out in 2022 using the search equation: “work engagement AND nurs^*^ AND burnout.” This search identified nine quantitative primary studies suitable for inclusion in our analysis.

**Results:**

Work overload, type of shift worked, and/or area of hospital service, among other elements, are all relevant to the development of burnout. This syndrome can be countered by social support and appropriate personal resources and values, which are all positively associated with work engagement. Our analysis revealed a significant correlation between work engagement and the different domains of burnout. The correlation effect size between burnout and work engagement was −0.46 (95% CI −0.58, −0.31), with *p* < 0.001.

**Conclusion:**

Well-targeted interventions in the healthcare work environment can reduce burnout levels, strengthen work engagement, and enhance the quality of healthcare.

## 1. Introduction

In nursing, highly complex tasks must be performed and decisions are taken in situations that are often difficult and stressful. Moreover, appropriate attention must be provided not only to patients but also to visiting family members. The working environment can include long days, rotating shifts, a severe emotional burden, and sometimes aggression expressed by patients, their families, or even colleagues (1). If this circumstance persists for an extended period of time, it can provoke fatigue, anxiety, distress, and discomfort, any or all of which may reduce the quality of care provided and heighten the probability of error. In such a case, there may be an evident imbalance between the human resources available and the demands placed on them (2).

The tasks performed by nurses are determined, on the one hand, by the day-to-day healthcare demands encountered, and on the other hand, by the resources available—personal, situational, and organizational. This balance is reflected in the job demands–resources model (JD-R) (3). Occupational demands may be physiological and/or psychological, and each type imposes a cost on the individual. In many situations, workers are subjected to stress and heavy workloads, although these may be counteracted by the application of situational resources (4), such as companionship, autonomy, opportunities to learn, institutional recognition, and the possibility of promotion, as well as personal resources such as resilience, self-efficacy, and optimism (5). Both types of resources help the professional adapt to the work environment, fostering the ability to cope (6), generating motivation and work engagement (WE), and thus reducing the probability of burnout (7, 8).

Burnout is a consequence of long-term harmful stress in the workplace. If the individual lacks resources to deal with this situation, the response made may be maladaptive and prolonged (9). Maslach and Leiter defined three domains of burnout syndrome: emotional exhaustion, depersonalization, and low personal accomplishment (10). It may be counteracted, however, by high levels of WE, that is, a state of satisfaction, commitment, and motivation. This, too, consists of three domains: vigor (effort, self-generated energy, and resolve), dedication (exaltation, empowerment, and active assistance), and absorption (immersion and high levels of concentration in the activity being performed) (11).

Traditionally, the concepts of burnout and WE have been considered opposing and independent. Paradoxically, however, some studies have observed the simultaneous presence of burnout (12) and WE (13–15), which suggests that these concepts, while independent, have a certain negative correlation (3, 16). Nevertheless, demanding occupational conditions are associated with burnout, while the availability of appropriate resources contributes to WE (16), although the beneficial impact of these resources varies according to the population considered and the environment in question.

As members of the multidisciplinary team in critical and emergency care services, nurses play a vital role in improving the quality of care and in reducing morbidity, mortality, and their associated health costs (17). Moreover, incorporating nurses into the multidisciplinary oncology team benefits the team's overall performance and can shorten the duration of treatment (18). Therefore, a good understanding of the working conditions experienced by nurses and other health workers will facilitate an organizational approach to help prevent burnout and foster WE, thus enhancing the care provided (19).

In summary, the aim of this study is to identify and analyse the factors that affect WE and burnout in nurses and then to determine the relationship between these reactions to the occupational environment.

## 2. Methods

### 2.1. Search strategy

A systematic scoping review was performed, in accordance with the PRISMA Extension for Scoping Reviews (20), based on data obtained from a search of the PubMed/MEDLINE and Scopus databases carried out in September 2022 using the Medical Subject Headings (MeSH search equation: “work engagement AND nurs^*^ AND burnout”).

### 2.2. Inclusion and exclusion criteria

The following inclusion criteria were applied:

- Primary full-text sources in English or Spanish.
- Quantitative articles with sample populations of nurses.
- Articles that measure the correlation between WE and burnout, or establish their predictive characteristics.
- Studies published between 2016 and 2022.

The exclusion criteria were as follows:

- Doctoral thesis.
- Articles with mixed samples without independent data on the nursing staff.
- Articles whose main objective is not correlated with the subject of study.

The selection of articles for analysis was carried out in three steps: First, the titles and abstracts were read, followed by a full-text reading of those remaining for analysis. Finally, a critical reading was conducted of each text finally selected, in order to assess the method applied and to detect any publication bias.

### 2.3. Level of evidence

The quality of the studies included in this review was assessed in accordance with the levels of evidence and degrees of recommendation stipulated by the Oxford Centre for Evidence-Based Medicine (OCEBM) (21).

### 2.4. Variables, data collection, and data analysis

The following data, obtained from each study/article, are summarized in Table 1: (1) author, year of publication, country of the study; (2) design; (3) sample; (4) results; (5) level of evidence (OCEBM); and (6) grade of recommendation (Table 1). A correlation effect size meta-analysis was calculated using StatsDirect software. Random effects were used for the calculation, and heterogeneity was assessed by i^2^. The Egger test was used to test for publication bias, and a sensitivity analysis was performed to check that none of the studies significantly affected the effect size.

**Table 1:** General information on the studies considered.

**References (country)**	**Design**	**Sample**	**Measuring instrument**	**Results**	**LE/ GR**
**(A)**					
Hetzel-Riggin et al. (22) (USA)	Cross-sectional study	76 nurses	- Nursing Stress Scale - UWES - Resiliency Scale - MBI	- Staff shortages caused significant problems regarding the quality of work experience (81%), the ability to maintain patient safety (71%), and the quality of patient care (78%). - The two most frequently reported stressors were lack of staff (30.9%) and excessive workload (28.4%).	2c/B
Mohamed et al. (23) (Egypt)	Cross-sectional study	280 nurses	- Mattering at Work Scale - UWES - MBI	−42.9% of the nurses studied had a moderate level of work commitment (physical, emotional, and cognitive) and 32.1% had a low level. 32% had a high level of burnout, and 35% had a low level. - A negative correlation was measured between engagement and burnout.	2c/B
García-Sierra et al. (24) (Spain)	Cross-sectional study	100 nurses	- JDRQ	- Social support is a significant predictor of WE. - WE moderates the relationship between labor demands and burnout syndrome. - The processes that influence WE and burnout are not independent.	2c/B
**(B)**					
Elst et al. (25) (Belgium)	Cross-sectional study	675 nurses	- SIMPH - QEEW	- Workload, work hours, and emotional demands are positively associated with burnout. - Labor resources are negatively associated with burnout and positively associated with WE. - Duration of employment in the service is negatively associated with WE. - Social support moderates burnout.	2c/B
Castro et al. (26) (Portugal)	Cross-sectional study	206 nurses	- MBI - DASS21 - Gallup Questionnaire	- There is a high frequency of burnout among critical care providers. - Stress, the number of working days, and the risk of burnout are interrelated. - There is a positive correlation between depression, anxiety, stress, and burnout. - There is a negative correlation between burnout and WE.	2c/B
**(C)**					
Sun et al. (27) (China)	Cross-sectional study	245 nurses	- QNWLS - MBI - UWES	- Quality of working life and WE have a positive impact on nursing and career identity. Job burnout plays an intermediary role in career identity. - There is a negative relationship between the quality of working life and burnout.	2c/B
Matziari et al. (28) (Greece)	Cross-sectional study	214 nurses	- FOCUS-9 Questionnaire	- Hospital nurses present higher levels of burnout and lower levels of WE than those in health centers. - Organizational values and support are positively associated with WE.	2c/B
Rosas-Paez et al. (29) (Mexico)	Cross-sectional study	56 healthcare professionals	- UWES-9 - MBI-HSS	- High and very high levels of WE were found in 55% of the sample. - A high level of WE is negatively correlated with burnout.	2c/B
Wan et al. (30) (China)	Cross-sectional study	245 nurses	- QNWLS - PINS - UWES - MBI	- There is a significant positive relationship between WE, satisfactory job characteristics, and an optimal work environment. - Age is significantly correlated with WE.	2c/B

DASS21, Depression Anxiety Stress Scale-21; GR, Grade of Recommendation; JDRQ, Job demands–resources Questionnaire; LE, level of evidence; MBI, Maslach Burnout Inventory; MBI-HSS, Maslach Burnout Inventory—Human Services Survey; PINS, Professional Identity in Nursing Survey; QEEW, Questionnaire on the Experience and Evaluation of Work; QNWLS, Quality of Nursing Work Life Scale; SIMPH, Short Inventory to Monitor Psychosocial Hazards; UWES, Utrecht Work Engagement Scale; WE, Work engagement.

## 3. Results

**Figure 1:**
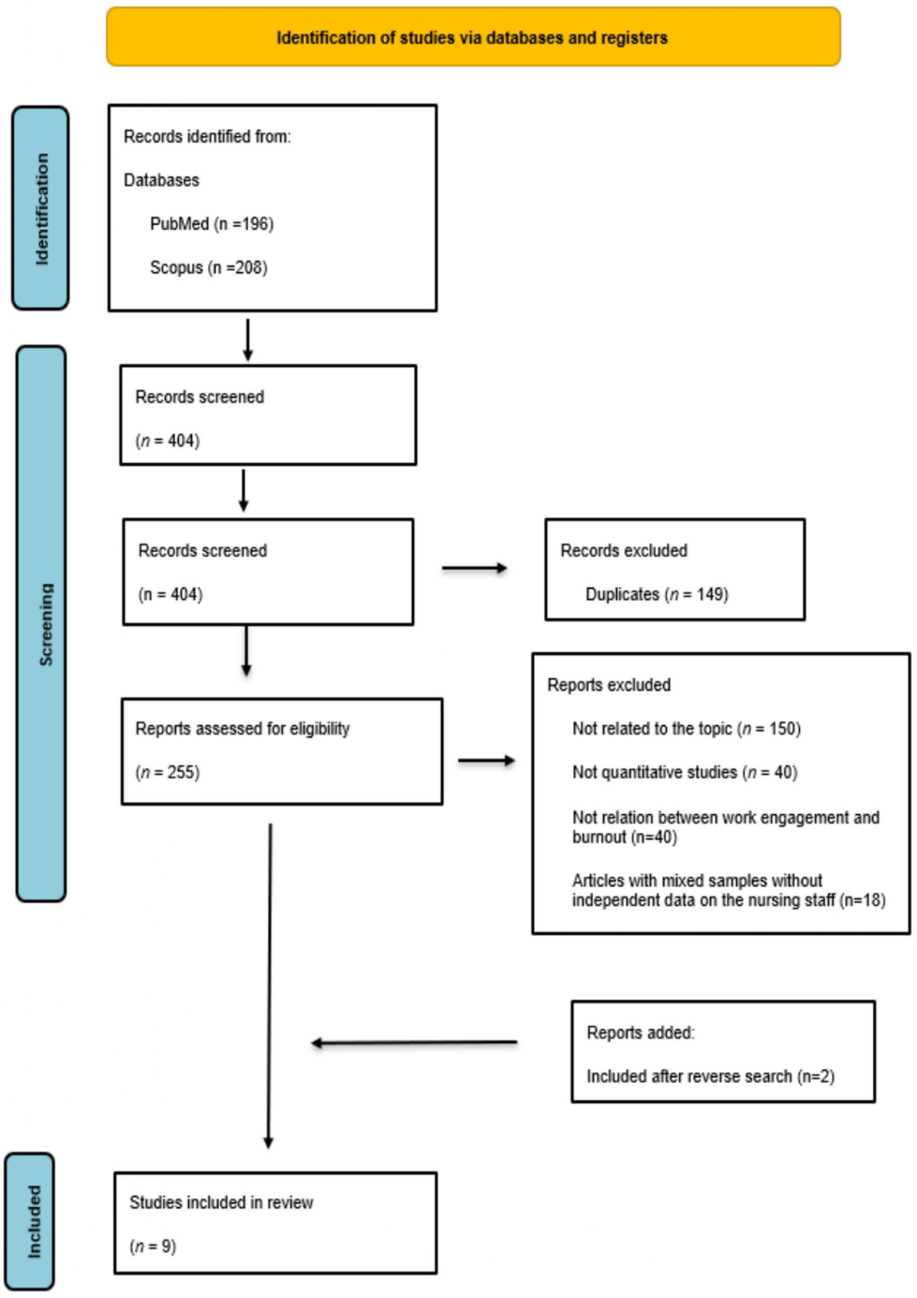
PRISMA flowchart of the study selection process. Adapted from Page et al. (31), licensed CC-BY-4.0.

The literature search obtained 404 articles. After excluding duplicates and applying the inclusion and exclusion criteria, 65 remained for full-text reading. This led to a further 58 being excluded, leaving seven for the final analysis review. In addition, a reverse search performed on these articles led to another two being included. Thus, nine studies were finally reviewed (Figure 1).

### 3.1. Burnout risk factors

Among the burnout risk factors for nurses, a positive correlation was observed between emotional exhaustion (a dimension of burnout) and workload. This dimension was also aggravated by emotional demands on the nurses. The longer the nurses had been employed in the service, and the more prolonged or unstable their working hours (for example, in the form of rotating shifts), the more likely they were to suffer burnout (25). Nurses who work in critical care units are more liable to suffer burnout than those working in other departments. Other factors that are positively associated with the presence of burnout syndrome include lengthy work schedules, anxiety, depression, stress, and problems with interpersonal relationships (26). Finally, a strong correlation between burnout and workload has been reported, with the latter being a significant predictor of the syndrome (26, 32).

### 3.2. Protective factors of work engagement

According to the studies analyzed, good organizational values and practices, together with support from co-workers and leaders, are positively correlated with the WE of healthcare professionals. Vigor and dedication, which both contribute to WE, are positively associated with the presence of clearly stated corporate objectives, rules, and procedures (28). Other factors that favor WE include control over one's work, decision-making powers and abilities, the development of professional skills, the perception of social support, and the existence of learning opportunities—in short, satisfactory job quality (25–27, 32).

Nurses working in surgical areas tend to present greater WE than those in emergency or medical care units. Moreover, hospital nurses often have lower levels of WE than those who work in health clinics (28). The specific characteristics of the job and the work environment, and even the age of the individual may also influence the development or otherwise of WE (30).

### 3.3. Correlation between work engagement and the dimensions of burnout

The dimensions of burnout considered were emotional exhaustion, depersonalization, and low personal accomplishment, while those of WE were vigor, dedication, and absorption.

Low levels of vigor are associated with a high risk of developing burnout due to emotional exhaustion (a perfect linear correlation has been reported in this respect). Burnout may also arise from low personal fulfillment, even if high levels of vigor are reported. Among the professionals who present low or very low dedication, there is a very high probability (90–100%) of burnout due to emotional exhaustion (23).

In addition, vigor is a significant negative predictor of emotional exhaustion, while absorption is a significant positive predictor of this condition. On the other hand, it is not significantly predicted by dedication. With respect to depersonalization, another dimension of burnout, none of the WE domains were significant mediators or direct predictors. Finally, dedication is a positive direct predictor of personal fulfillment, but neither vigor nor absorption is a significant predictor in any sense (22, 23).

According to several studies, WE is most frequently (around 50% of cases) classified as moderate, in terms of the three domains considered (vigor, dedication, and absorption). The correlation between WE and burnout is reported to be negative and highly significant (22, 23, 27, 29).

### 3.4. Meta-analysis of the correlation between burnout and work engagement

**Figure 2:**
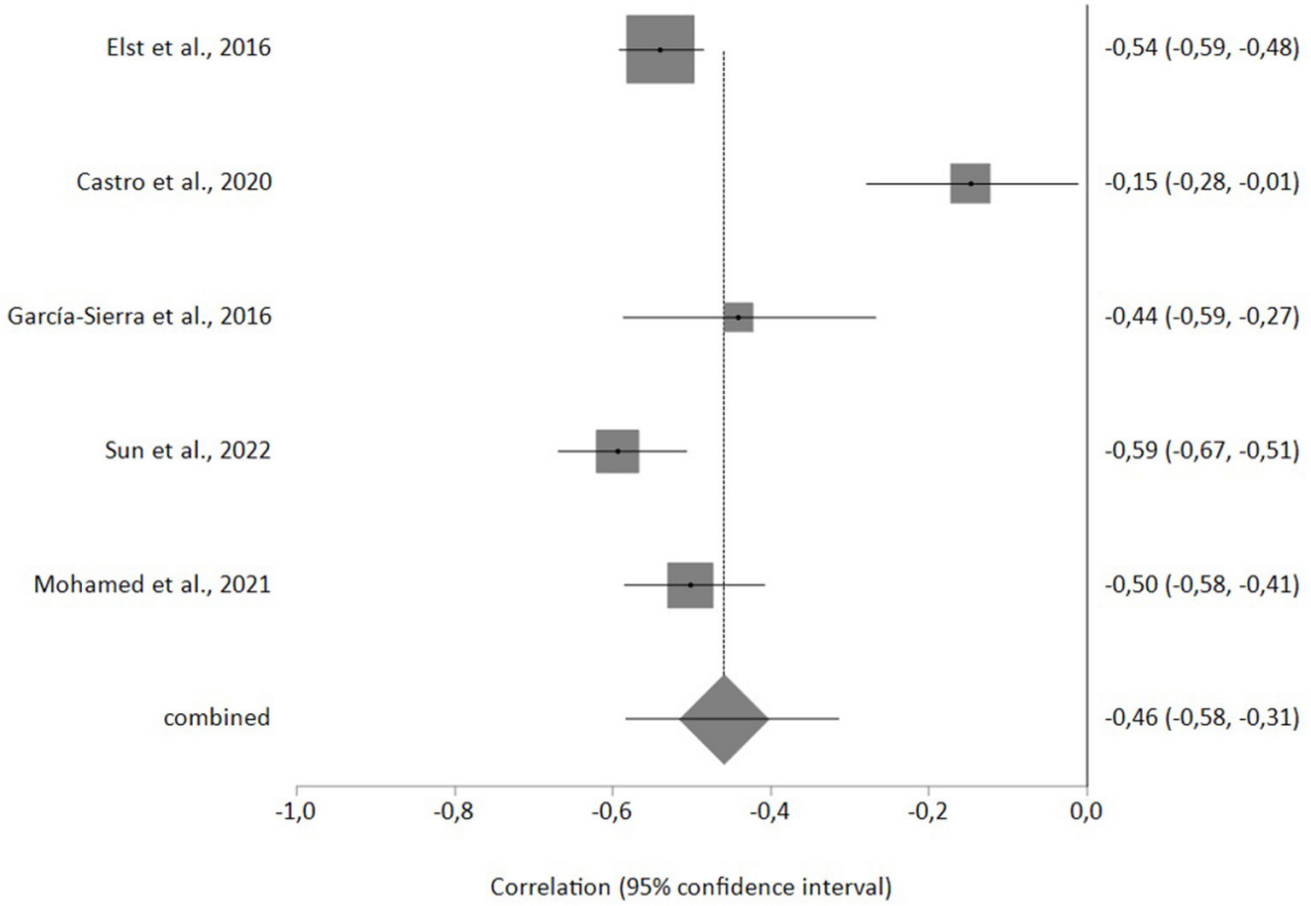
Forestplot of the correlation between burnout and work engagement.

Five studies were included in the meta-analysis, with a total sample size of 1,506 nurses. The correlation effect size between burnout and WE was −0.46 (95%CI −0.58, −0.31) with *p* < 0.001 and the heterogeneity (i^2^) was 89%. The Egger test did not reveal any publication bias, and the sensitivity analysis did not suggest any publication that had to be excluded (Figure 2).

## 4. Discussion

This study aims to enhance our understanding of how personal characteristics and workplace-related factors may influence WE and trigger the presence of burnout among nurses. Given this information, appropriate measures can be adopted to reduce the risk of burnout and to foster greater participation and commitment by these workers.

Our analysis shows that nurses' WE is favored by environments in which they have autonomy of decision-making, where they are given sufficient resources to carry out their work, and where there is greater altruism among workers (33). In other words, WE benefits from a strengthening of the nursing identity, which generates pride in job performance and enhances the working experience (34). In turn, higher levels of support among nurses will decrease the burnout experienced (33, 35). The hospital area in which a nurse work is also relevant to WE and job satisfaction, which are both affected by the emotional impact or workload experienced in different areas of health care (36). According to previous research, when WE is high, nurses are less likely to request a transfer to a different work unit or service. Moreover, favorable occupational health conditions are expected to improve relationships and collaboration among workers, which enhances the care provided, and thus generates a positive gains spiral (24).

Among the factors found to increase the risk of burnout is the hospital area in which the nurses work. Those employed in an Accident and Emergency (A&E) department are more likely to generate a psychologically distant relationship with patients, as a consequence of the high level of burnout experienced (16). This, in turn, can cause patients to have negative perceptions of the quality, effectiveness, and efficiency of the healthcare received (37, 38). Another relevant factor is the type of work shift performed; thus, nurses who work night shifts have been shown to present higher levels of burnout. In short, professionals who undergo high levels of stress and fatigue are more liable to present detachment and dysfunctional attitudes (39). Burnout can also be caused by lengthy employment in the same medical unit/service. In response, and seeking to alleviate this condition, long-standing workers might request to be transferred elsewhere (40). In addition to the above, many other elements can influence the appearance of burnout syndrome among nurses, such as the female stereotype of the profession, the lack of recognition of the ‘invisible' tasks performed, and an excessive level of bureaucracy, as pointed out by Manzano Garcia et al. in their qualitative e-Delphi study (41). These questions have received little previous research attention.

Finally, consideration should be given to the COVID-19 pandemic, which has had a negative impact on the mental health of critical care nurses and their families, as public safety considerations have been prioritized over those of patient care. This situation has generated great unhappiness among the nurses affected, despite the support received from friends and colleagues (42, 43).

After considering the factors relevant to burnout and WE, we then determined the relationship between them, taking into account the three dimensions of each. Our analysis confirms previous findings that there is indeed a close relationship between the two concepts, and specifically that greater WE (as concerns each of its constituent elements) reduces the risk of burnout (40, 44).

A lack of vigor is associated with higher levels of emotional exhaustion. This is in line with the Utrecht Work Engagement Scale, according to which there is a strong interaction between these two dimensions (45). Therefore, the goal of recognizing WE among nurses and establishing strategies to promote it may be limited by the presence of emotional exhaustion (46). Conversely, dedication is inversely related to the presence and impact of burnout. Some of the studies in our analysis concluded that professionals who were unable to perform the work expected of them, or who were unable to meet the needs raised, whether by the patient or by the organization, due to decreased levels of dedication and perceptions of insufficiency, were at a high risk of developing burnout (47). In this respect, too, some authors indicate that a feeling of low personal fulfillment may arise from the view that the tasks performed are not considered important or productive. This impression would tend to reduce dedication and hence WE (48). Finally, high levels of absorption might provoke burnout, if this absorption prevents the worker from achieving an objective emotional balance (37, 40).

Our study is subject to certain limitations, which should be acknowledged. First, by restricting the articles considered to those published in English or Spanish, we may have omitted potentially significant research findings from studies published in other languages which otherwise met the inclusion requirements. Furthermore, the studies included did not all use the same measurement instruments, so the results presented may not be homogeneous. Finally, the relationship between burnout and WE has only recently been the object of academic study, and so relatively few articles are available for consideration.

## 5. Conclusion

The correlation between low WE and burnout in nurses not only impacts these professionals but also has damaging consequences for patients and the health system in general.

The studies considered in our review describe varying degrees of burnout and WE. The relevant factors identified include employment conditions (such as work overload and type of shift or service area), personal characteristics (such as perceived support and the individual's own values), and organizational resources. All of these factors influence WE and hence the possibility of burnout.

The results obtained from our analysis highlight the need to design and implement effective interventions in the workplace in order to address the problematic areas identified and thus reduce the risk or degree of burnout among nursing personnel. This, in turn, would enhance levels of WE. Failure to do so might be damaging not just for the workers concerned but also for the quality and safety of public healthcare.

As a final observation, further studies in this area are needed in order to better understand the phenomenon of burnout among nursing staff.

## Data availability statement

The original contributions presented in the study are included in the article/Supplementary material, further inquiries can be directed to the corresponding author.

## Author contributions

MAV-B, FJI-E, and GAC-F: conceptualization. FJI-E, AV-S, and LA-G: data curation. NC-M, JLG-U, and AV-S: formal analysis. MAV-B, JLG-U, and AV-S: investigation. AV-S, JLG-U, and GAC-F: methodology. MAV-B and AV-S: resources. JLG-U: software. LA-G and GAC-F: supervision. JLG-U, NC-M, and GAC-F: visualization and validation. MAV-B, NC-M, and FJI-E: writing—original draft, review, and editing. GAC-F: funding acquisition and project administration. All authors contributed to the article and approved the submitted version.
